# Challenges of Large-Scale Multi-Camera Datasets for Driver Monitoring Systems

**DOI:** 10.3390/s22072554

**Published:** 2022-03-26

**Authors:** Juan Diego Ortega, Paola Natalia Cañas, Marcos Nieto, Oihana Otaegui, Luis Salgado

**Affiliations:** 1 Vicomtech Foundation, Basque Research and Technology Alliance (BRTA), 20009 San Sebastián, Spain; pncanas@vicomtech.org (P.N.C.); mnieto@vicomtech.org (M.N.); ootaegui@vicomtech.org (O.O.); 2 ETS Ingenieros de Telecomunicación, Universidad Politécnica de Madrid (UPM), 28040 Madrid, Spain; 3 Grupo de Tratamiento de Imágenes, Information Processing and Telecommunications Center and ETS Ingenieros de Telecomunicación, Universidad Politécnica de Madrid (UPM), 28040 Madrid, Spain; luis.salgado@upm.es

**Keywords:** ADAS, driver monitoring, multi-camera, automotive, datasets

## Abstract

Tremendous advances in advanced driver assistance systems (ADAS) have been possible thanks to the emergence of deep neural networks (DNN) and Big Data (BD) technologies. Huge volumes of data can be managed and consumed as training material to create DNN models which feed functions such as lane keeping systems (LKS), automated emergency braking (AEB), lane change assistance (LCA), etc. In the ADAS/AD domain, these advances are only possible thanks to the creation and publication of large and complex datasets, which can be used by the scientific community to benchmark and leverage research and development activities. In particular, multi-modal datasets have the potential to feed DNN that fuse information from different sensors or input modalities, producing optimised models that exploit modality redundancy, correlation, complementariness and association. Creating such datasets pose a scientific and engineering challenge. The BD dimensions to cover are volume (large datasets), variety (wide range of scenarios and context), veracity (data labels are verified), visualization (data can be interpreted) and value (data is useful). In this paper, we explore the requirements and technical approach to build a multi-sensor, multi-modal dataset for video-based applications in the ADAS/AD domain. The Driver Monitoring Dataset (DMD) was created and partially released to foster research and development on driver monitoring systems (DMS), as it is a particular sub-case which receives less attention than exterior perception. Details on the preparation, construction, post-processing, labelling and publication of the dataset are presented in this paper, along with the announcement of a subsequent release of DMD material publicly available for the community.

## 1. Introduction

Data has become the most important asset in the development of advanced driver assistance systems (ADAS) and autonomous driving (AD) functions. Deep learning (DL) and other artificial intelligence (AI) methods are being employed in the development and testing phases of many ADAS/AD functions present in modern vehicles [1]. Their non-deterministic nature obliges the industry to shift closed-loop validation approaches into long-term Big Data procedures, where sensor data fuels training and testing processes [2].

Large volumes of carefully crafted sensor data are needed for two main purposes: (i) to create datasets as input for DL training mechanisms and (ii) to create ground-truth metadata for validation of ADAS/AD functions in SiL (software-in-the-Loop) or HiL (hardware-in-the-Loop) setups.

The more complex the information the AI or function produces, the larger and richer the datasets needed. The evolution of open datasets in the context of automotion shows the trend to have multi-purpose and multi-sensor (e.g., LIDAR and cameras) datasets, which can be used both for training and validation of functions. The production of such datasets is no longer feasible for small companies or university groups. Compared with small-scale datasets, a large-scale dataset implies a tremendous technical and economical effort for designing, setting up, recording and post-processing data according to the quality standards required by the AI frameworks and the automotive industry.

Most of the largest public datasets in the ADAS/AD domain focus on exterior perception systems, ideal for building automated braking systems, lane departure warning methods, etc. However, there is a clear lack of datasets centred on monitoring the interior of the vehicle, i.e., the driver. Excluding SAE-L5 autonomous driving, which is still not a reality, the driver is still a key figure in the driving task. Though, as automated levels increase, there is a continuous shift in humans’ role from driver to passenger, which can occur during driving situations, especially when automated functions get out of their defined operational design domains (ODD). In such situations (e.g., exiting from the highway), a transition between automated and manual driving modes must happen respecting safety regulation and principles. In that sense, driver monitoring systems (DMS) have gained focus in the industry. Commercial vehicles have incorporated some sort of driver attention systems since 2006 [3,4,5], analysing driving patterns and behaviour. However, camera-based DMS has emerged as the most unobtrusive technology that enables gathering information about the physiological state of the driver: fatigue, behaviour and distraction, without interfering with the driving task itself or implying a decrease in comfort.

Building AI-based DMS has, therefore, become a relevant topic for the automotive industry and scientific communities. Despite having similarities with exterior perception systems, DMS’ subject of analysis is humans in the interior of a small cabin performing driving tasks along with other actions (talking to passengers and interacting with car controls or other devices). The aim of DMS is to produce data that assesses the ability of the driver to perform the driving tasks and to monitor the driver’s state at all times. As a consequence, DMS data is complex (fatigue and distraction related), heterogeneous (facial features and actions), human centred (individuals may show different behaviour patterns) and mostly centred in camera sensors.

Datasets for DMS need to satisfy several requirements:

- Large amounts of data for training and validation of DL methods.
- Realistic data of relevant situations (e.g., safety related such as drowsy drivers, talking on the phone, etc.).
- Spatio-temporal labels, including visual features (e.g., face landmarks) but also actions as frame intervals with a semantic load.
- Captured data need to represent physiological states for fatigue, behaviour and distraction, and thus several cameras might be needed to monitor the face, hands and body of the driver.

The preparation of a DMS dataset that meets these requirements imposes a number of important challenges, most of them from a technical perspective but also economical and organisational:

- Preparation of multiple environments (real car and simulator for simulation of non-safe driving behaviours or physiological states).
- Creation of complex annotation or metadata schemes to host heterogeneous labelling data.
- Organisation of recording sessions and management of volunteers.
- Data preparation: recording, transferring and compressing large volumes of raw data.
- Data processing: synchronization, alignment, calibration, labelling, etc.
- Privacy and ethical aspects (GDPR compliance).
- Dissemination aspects, including website preparation, management of updates, GitHub repositories, user manuals, samples, etc.

In this paper, we overview the challenges faced to build a multi-camera and multi-modal large-scale dataset for DMS. The manuscript describes the approach to solve the technical and organisational issues that appear during the design and construction of DMS. The main contributions of this work are:

- Definition of a multi-sensor set-up architecture (multi-modal and multi-camera) for capturing large-scale video footage in the context of driving monitoring.
- Organisational approach to manage human volunteers and recording sessions: environments, scripting, recording, privacy and ethical aspects, etc.
- Orchestration of data preparation and data processing stages considering the DMS requirements: storage, containers, transmission, compression, synchronization, alignment, calibration and labelling.
- Taxonomy of driver monitoring labels for multi-level dataset annotation.

The reported work can serve as a reference approach to create other multi-camera and multi-modal large datasets for interior or exterior monitoring applications in the automotive domain. This methodology was applied to build the recently published DMD (driver monitoring dataset) in the context of the activities initiated during the execution of the European project VI-DAS. The results of the work include the creation of recording and labelling tools, the implementation of new methods to synchronize and align streams and the construction and publication of the DMD, one of the largest open datasets designed to develop DMS.

The paper is organised as follows: Section 2 overviews other works on large-scale dataset creation. In Section 3, the design of the dataset and architectural principles of the recording set-up are presented, while Section 4 describes the post-processing steps. Discussion around the challenges faced and proposed solutions is presented in Section 5.

## 2. Driver Monitoring Methods and Datasets

### 2.1. Datasets in the Automotive Sector

The main objective of the DMD is to gather a diverse collection of image data of the interior of the vehicle based on the most relevant features to observe inside the cabin. The target is to provide driver monitoring practitioners with a semantically rich dataset that allows the design and generation of DL-based algorithms for driving monitoring.

The revolution of DL methodologies [6] has made indispensable the availability of large volumes of high-quality data to train network models. To extract all the potential of DL techniques for computer vision tasks, the available data need to have a diverse collection of annotations aimed at tackling different tasks. By using these annotated datasets and a variety of state-of-the-art DL methods [7,8,9,10,11], a DL neural network can then be used and fine-tuned for the particular application in the driving context. Many generic datasets have been proposed: ImageNet [12], MS Coco [13] and PASCAL [14,15]. All of these datasets consist mainly of a collection of images containing instances of different objects with their corresponding label to address the object detection task in scene understanding. The application of these datasets is limited to generic object detection and semantic segmentation tasks, where the object can appear in a wide range of scenarios.

Although these datasets can be the starting point for training object detection models, the adaptation to the automotive domain results in limited and not robust DL models. Hence, additional effort is required to adapt and fine-tune the models by using domain-specific data. In addition, such types of datasets only provide information from one type of sensor: an image camera. Having only one modality of input data may limit the robustness of autonomous systems in complex scenarios.

In order to adapt the DL models and provide multi-modal data in the context of automated driving, there has been an intense development and effort to design, build and make available large-scale datasets to sense the exterior of the vehicle. Both industry and academia have published diverse collections of data that combine RGB images, IR images, LiDAR point clouds and high precision GPU/IMU inertial navigation data. Some of the first to appear and most relevant datasets for exterior perception include: KITTI [16,17], WAYMO [18], Cityscapes [19], nuScenes [20] and Lyft Level5 [21].

The experience gathered to produce these massive datasets for exterior sensing has resulted in some common practices in terms of the type of data and sensors used for recording the sessions. Based on some shared processing tasks such as: object detection (pedestrian, vehicles, obstacles, etc.), segmentation (free space and instances), 3D sensing or localization and positioning; these datasets have defined the path to produce a large-scale dataset for analysing the exterior of the car. However, the level of maturity in the generation of databases focused on driver monitoring has not been reached yet, even though there have been some efforts to produce datasets aimed to cover specific tasks of driving monitoring.

### 2.2. Driver Monitoring Systems and Data Requirements

The driver’s behaviour involves different body and face activities. Ensuring normal driver behaviour is critical to avoid any human error that could eventually produce traffic accidents with the consequent harm of driver and passengers. Therefore, to prevent vehicle accidents, systems are built to monitor the drivers and assess their arousal and distraction level. Among driver behaviour conditions, drowsiness and distraction are the two most relevant subjects of study when designing DMS [22]. According to the type of input data provided by the measurement sensor, systems designed to analyse and assess driver behaviour can be broadly divided into two categories: visual features based and non-visual features based.

Techniques based on non-visual features obtain the input data from sensors, which can either be intrusively attached to the driver’s body [23] or require the extraction of car parameters [24]. Within this group, techniques are also divided into two categories: *driver physiological features analysis* and *vehicle parameter analysis*.

Physiological features are often a good indicator of early signs of fatigue or distraction. Sensors to measure physiological features of the driver extract signals from the person’s heart, brain, eyes or skin. Changes in physiological signals such as electroencephalography (EEG) [25], electrocardiogram (ECG) [26], electro-oculography (EOG) [27] and surface electromyogram (sEMG) [24] can be an accurate method to detect driver state. Physiological features are a direct measure of fatigue, however, their application in real driver fatigue and distraction detection systems is limited due to intrusive characteristics of the sensors used and critical issues to eliminate noise and artefacts, which are inevitable in real-world driving conditions.

Moreover, it is well known that fatigue and distraction reduce the driver’s ability to perform. Therefore, other researchers have studied parameters obtained from the vehicle sensors to analyse the driver’s state. The deviation in features such as lane crossing [28] and steering wheel angle [29] are indicators of deteriorating driving ability. Other strong indicators of abnormal driver behaviour also include unusual activities such as pressure changes on brake and accelerator [30], load distribution on the driver’s seat [31] and vehicle speed [32]. In general, there is limited research available exploiting this technique. Vehicle signals for driver inattention assessment are easily affected by driving habit, driving skill, vehicle speed, vehicle type and road conditions. Therefore, a robust DMS solution based only on vehicular signals is not yet feasible.

Techniques using visual features usually rely on the use of camera sensors pointing to the driver to obtain input data. Image processing techniques enable monitoring of these activities through a camera capturing the most relevant parts of the driver. Image-based DMS should address two primary tasks: analysing the sequence of images for extracting important indicators and evaluating the temporal evolution of driver state using such indicators.

Recent DMS has focused on evaluating different driver states using visual-based camera sensors. RGB or visible spectrum cameras, depth cameras or infrared cameras are the three main types of sensors used in vision-based DMS. Among the different methods and systems found in the literature, RGB cameras are the most applied in research DMS [33]. The reason is that visible spectrum cameras are affordable and their integration in modern systems is easy to implement. However, due to illumination limitations in real driving conditions, NIR external illumination and IR camera sensors are preferred since the use of DMS can be extended to low-light conditions [34,35]. Other works have integrated depth sensors to provide additional data to the DMS algorithms and better predict driver state [36]. Besides visual data, some efforts have been conducted to incorporate other modalities such as audio using microphones attached to the person to estimate stress and emotions in drivers [37]. The outcome of many of the currently available DMS methods are pushing forward to fuse several modalities and data streams to develop a robust method to infer driver state [38,39].

### 2.3. Datasets for Application of Driver Monitoring Systems

**Figure 1:**
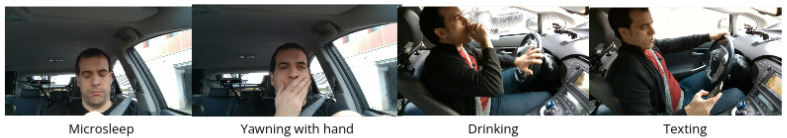
Examples of activities performed in the DMD.

Most of the work to generate visual datasets was devoted to sensing and capturing the exterior of the vehicle. Although there are several initiatives that provide vision-based datasets for DMS, the lack of consolidated datasets has motivated our efforts to contribute with the creation of the DMD; in Figure 1, there are example images of some activities included in this dataset. Driver monitoring requires the interpretation of the driver’s features regarding the attention and arousal state, the direction of gaze [40], head pose [41], the position of the hands [42], blink dynamics [43], facial expressions [44], body posture [45] and drowsiness state [46]. The currently available datasets tackle these DMS dimensions individually and do not provide a general description of different driver conditions.

In Table 1, a list and description of the most relevant open datasets found at the moment are presented. The analysed datasets present data of the driver focused on specific parts of the body such as the face [47], hands [42,48] or upper body [49]. The sensors’ types are primarily RGB cameras and depth sensors. The metadata labels available in these datasets are varied and include geometrical features such as bounding boxes, landmarks, masks and features such as temporal actions. Moreover, the scenarios used to record the data differ between real vehicles and in-lab simulators. Most of the datasets only include one camera to perform the recordings. The recent dataset in [50] provides visual data from different points of view using several cameras. However, their target is the analysis of driver actions during autonomous car functions.

Based on the limitations of the currently available in-cabin driving monitoring datasets, the limited size of these datasets and the increasingly new challenges and requirements of DMS, the DMD project has built a preparation methodology to collect different sources of drivers’ behaviour data and deploy the required metadata annotations to be used by different driving monitoring sensing systems.

## 3. Dataset Definition and Creation

The preparation of a large-scale dataset for DMS involves different organisational and implementation stages, which require careful planning and execution. During the preparation of the DMD, three stages were defined. The first stage involved the preparation of the resources needed and planning the execution of the recordings. During the second stage, the actual recordings with the volunteers were performed. The third stage consisted of post-processing the recorded material, data extraction in compressed and manageable formats, solving syncing and alignment issues between video streams and annotation of the defined classes and labels.

**Figure 2:**
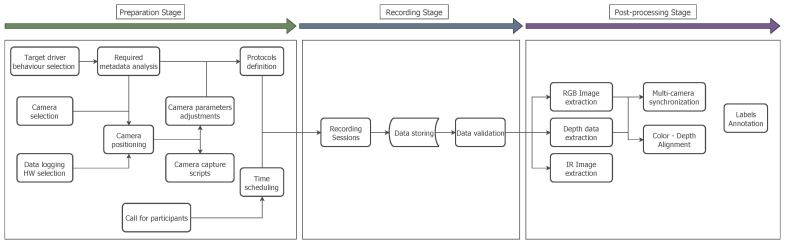
General overview of the DMD creation process.

In Figure 2 the complete process for the creation of the DMD is depicted, showing the three main stages for creating the dataset and the sub-actions for each stage. The relation between the tasks and the timeline required to complete all the required actions is also represented. Some of these steps can be performed in parallel to reduce the time spent in the creation of the dataset.

### 3.1. Metadata Taxonomy Definition

Planning the creation of a computer vision dataset requires having in mind the target type of annotations to be exported. A rich and varied set of labels makes the dataset more usable for different scenarios and problems, especially when the task to be solved can be decomposed as a set of small jobs.In particular, the implementation of DMS usually involves the combination of task-specific algorithms with the final objective of exporting a set of features to describe the actual state of the driver [55].

The required target metadata for the DMD was defined based on the analysis of the most relevant characteristics found in the state-of-the-art methods for DMS. Specifically, the DMD wants to cover three main pillars of DMS: drivers’ distraction detection, fatigue estimation and gaze estimation. The recordings and the annotation planning are oriented to support these methods; however, due to the DMD’s richness and variety of data, other uses of the dataset may appear in future studies.

Previous works have analysed driver distraction from still images, with techniques such as body pose estimation, which requires body landmarks and body position annotations [33]. Besides geometrical metadata, our goal was to perform the analysis including the temporal dimension; this involves recording moving sequences and providing temporal annotations. The head/face-centred analysis, for driver fatigue estimation for instance, typically focus on quantitative measures such as PERCLOS, eyes aperture, etc., which are calculated based on face landmarks. This implies the annotation of these spatial landmarks with the intention of also carrying out a temporal analysis.

**Figure 3:**
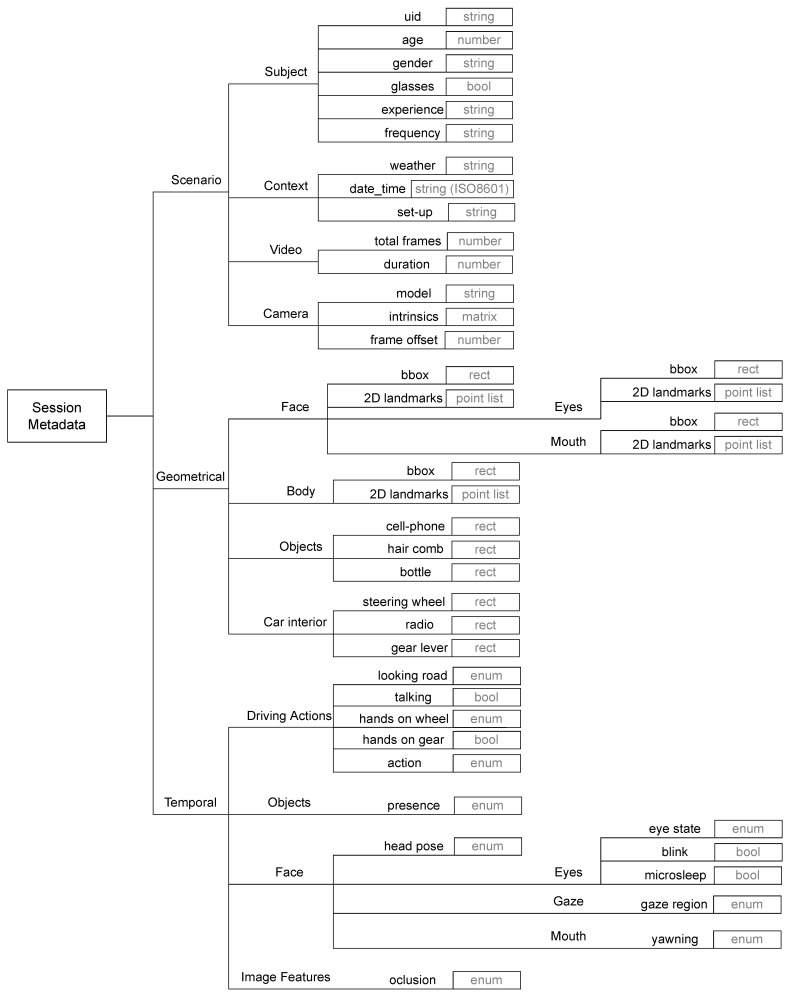
Metadata label taxonomy for the DMD.

To better distribute the dataset annotation tasks, we have defined three metadata groups: scenario, geometrical and temporal data. These groups allowed us to design the recording protocols and annotation guidelines, reducing ambiguity and boosting the value of the recorded material. The proposed taxonomy of features in the DMD is presented in Figure 3. This taxonomy is flexible and allows the inclusion of other labels within each category. Moreover, it is possible to increase the level of detail of the presented label tree.

#### 3.1.1. Scenario Metadata

The process of creating a vision-based dataset requires checking not only the availability of a diverse set of visual labels, but also other information that describe the capturing conditions of the data. For the creation of the DMD, we have taken into account this context information to complement the other visual labels, which are valuable for the development of robust DMS. Within this group, we have included information related to the driver (age, gender, use of glasses, driving experience, etc.), capture context (weather, timestamp-ISO8601 and capturing set-up), recording metadata (total number of frames, duration of session) and camera parameters (model, calibration and synchronization parameters). This scenario information is required to further analyse the correlation of some driver behaviour performance based on personal characteristics.

#### 3.1.2. Geometrical Features

For any computer vision dataset, geometrical labels are the core part of the dataset. Therefore, the DMD was designed to produce a diverse set of geometrical characteristics of the driver and the car interiors. The geometrical annotations considered within the creation of the DMD depict the visible characteristics of the scene related to the driver’s behaviour while driving.

The regions of interest where DMS typically focus are the face and body of the driver, mainly because it is possible to infer the actual state of the driver in terms of fatigue and distraction. For instance, fatigue is generally observed from eye activity, such as analysing the blinking cadence and frequency and computing some statistics that highly correlate with drowsiness and fatigue. To collect these types of features, we used a multi-sensor camera set-up for capturing the DMD and collecting information on the driver from different perspectives.

Geometrical features are usually represented as a set of values that encloses some spatial region. These features can be: (i) points such as image landmarks; (ii) bounding boxes to delimit a rectangular area such as the face or body of the driver; (iii) pixel masks to delimit a region of the images taking into account the object’s contour.

#### 3.1.3. Temporal Features

A temporal label describes a sequence or action through time. It assigns a semantic value, e.g., a name or type of action, to a time interval of a recorded video, indicating when the action begins and ends. A temporal annotation job starts with the definition of the semantic values or labels and the level of detail to be handled in the final annotations. For example, a general annotation should have one label that describes the entire sequence, such as ‘texting’; at a higher level of detail, this label can be derived into two, ‘texting left’ and ‘texting right’, differentiating the hand with which the person is texting.

Labels can be created and organised hierarchically (according to level of detail) and be logically related. In this way, it can be associated that, when the person is performing the ‘texting left’ action, which is sending a text message with the left hand, the person cannot be performing the ‘texting right’ action, that is, sending a message with the right hand. This analysis is important to consider the context in which the annotation task is performed to establish these logical relationships.

There are not only mutually exclusive labels which can not coexist in the same time interval. A sequence can be described by several labels simultaneously. To make this possible, they can be organised by levels. A level of annotation is composed of one label or a group of labels that are mutually exclusive.

Suppose the case of a person sending a text message and discriminating the hand with which he/she is holding the mobile phone; furthermore, you want to describe where the driver is directing his/her gaze while performing this action, either at the mobile phone or somewhere else. For this case, there should be two levels of annotation, the first one is to know how the person is performing the action of texting and the second level to know if the person is looking at the mobile phone or not. Those two levels of annotations have mutually exclusive labels since the person is either using the right or left hand to text and because his/her gaze is either directed at the phone or somewhere else, none can occur at the same time.

Using this logical and hierarchical organisation by levels, we created the annotations of the DMD. As presented in Table 2, we show an example of 7 levels of annotation for distraction detection purposes. All levels are mutually exclusive or encompass only one label.

It is crucial to be as descriptive as possible to avoid misinterpretations by the annotators. To begin with, the label text or name should be clear and specific: it must represent the sequence correctly and be different from other labels. It is also recommended to write a complete definition of each label, indicating when the sequence it represents should begin and end.

### 3.2. Scripted Protocols

There are several approaches to perform a driver monitoring task. This dataset focuses on three particular aspects: distraction, drowsiness and gaze direction. Therefore, the DMD planning included the capture of specific material of drivers in distracting situations, with signs of drowsiness and staring at different regions inside the car.

Driver behaviour is very complex and is conditioned to certain circumstances. To intentionally capture those behaviours, it is ideal to recreate the trigger situations to have data as realistic as possible. However, to collect realistic situations of the interesting driver behaviour, it is required to collect many hours of video and filter the relevant parts of the scene. In addition, sometimes some situations are very difficult to collect from naturalistic recordings, such as sleepy drivers, since these edge situations will put the driver’s safety at risk. Therefore, for situations where it is hard to collect data, the behaviour should be acted in the most natural way possible.

In order to avoid these limitations and for the sake of optimizing recording sessions, we propose the definition of a series of protocols that guide through the process of recording the desired driver behaviour. Despite the importance of spontaneity in the act of driving, the activities, times, conditions and states of the driver must be planned to meet the objective of content to be captured.

The recording of these protocols were scheduled at different days and times in the day so that there would be variation in lighting and weather conditions. In addition, the volunteer drivers were recorded several times, so different clothing were used during the recordings. A total of 150 sessions were planned and executed for this round of recordings. From this, a total of 87 were recorded in the morning and 63 were recorded in the afternoon time slot.

Each protocol contains a detailed instruction list of how to perform each experiment (recording session). This is ideal to maintain uniformity among experiments and ensure completeness and quality of content. Consequently, there are different protocols: one per each aspect of the driver’s analysis and per recording location (car or simulator). The general structure of a protocol has the following stages:

- **Participant welcoming:** Check which participant is programmed, call him/her and welcome him/her. A description of the context of the experiment must be explained to the participants, along with the legal privacy terms and a brief description of their function: perform the indicated activities in the most natural way while driving (real or acting) and if required with a certain state (drowsy or attentive).
- **Rehearsal and technology check:** A quick practice of the activities to perform is necessary, both to verify that the participant has understood the activities to perform and that the recording equipment (cameras, microphones, etc.) is working correctly.
- **Recording activities to perform:** The actual recording is carried out following a predefined list of actions to be performed or acted by the driver, specifying the time (seconds) that each activity should last, approximately. The person in charge of carrying out the experiment is the person who controls these times and indicates to the participant what action and when to perform it.
- **Final check:** It is proposed to conduct a quick check of the recorded material to verify that the recording session went well and prepare everything for the next participant.

#### 3.2.1. Distraction Protocol

The objective of this scripted protocol is to capture a person performing several activities related to the concept of distraction while driving. The list of proposed activities is presented in Table 3. When the driver is not distracted and is focused on the road or when the subject is not performing any of the activities related to distraction, it is considered ‘safe driving’.

Texting, phone call, hair and makeup and reaching behind are activities that are considered dangerous and illegal to perform while actually driving. This limitation motivates our use of a driving simulator that allows recording those activities while driving without the mentioned risks. For keeping the environment of an actual car, these dangerous activities were also captured with the car stopped. However, some activities such as operating the radio, talking to the passenger and reaching behind are not possible to record in the simulator because of its limitations of space and lack of adjacent real car elements.

The distraction protocol was then adapted into two parts: one exclusively for the activities included in the simulator recordings and another for those performed in the car. This last one was also divided into two sections: one for the activities executed with the car moving and one with the car stopped.

The estimated time to carry out one complete recording session of the distraction protocol in the car is 28 min per person, considering that first the protocol with the car moving is performed, followed by the one with the car stopped. For the simulator, a separate session is scheduled and it takes 20 min to complete its distraction protocol.

The footage approximate mean length in the simulator is 9 min per recording, 4 min for the stopped-car sessions and 8 min with the car moving.

#### 3.2.2. Drowsiness Protocol

In contrast with the distraction activities, drowsiness sessions, as shown in Table 4, are more complicated to achieve naturally, mainly because a state of drowsiness cannot be induced immediately; it has to be acted. Subjects were asked to show signs of drowsiness such as yawning, nodding and having micro-sleeps; although they are let free to perform the activities as they prefer to not affect the naturalistic behaviour of the action, it highly depends on the imitation abilities of the driver for these to seem natural. At the beginning, the participant is asked to drive normally, this can be manifested by an alert disposition for driving, checking the rear mirrors, firmly holding the steering wheel, etc. Then, the participant imitates sleepy driving, which is characterized by a tired position of the head and body, a certain lack of attention to the surroundings, short eyelid aperture and occasional nodding. It was indicated to the driver when to perform the signs of drowsiness. He/she maintained this sleepy driving behaviour throughout the recording until its completion.

It took approximately 10 min for every execution of the drowsiness protocol, and the length of the resulting recordings is about 3 min per session and setup.

#### 3.2.3. Gaze and Hands Protocol

In this protocol, 9 regions of gaze direction were established. These are zones inside the car where the driver frequently looks while driving and which are wide enough to be considered a region. These same regions were also calculated in the simulator. The simulator’s screens position and rotation are arranged to fit a realistic car cabin in terms of perspective. This produces the feeling of being in a vehicle and allows maintaining the same regions to detect gaze direction. The participant was asked to stare at each of the regions for 5 s when indicated.

In addition, for this protocol, we also established an analysis of the driver’s hand position. The objective of defining a set of actions is to gather a diverse collection of hand positions while using the steering wheel. During the sessions performed using this protocol, the driver adopts one position for a few seconds and then changes to the next when indicated. To add variation, the hands were captured in two situations: standing still and moving as if the participant was driving.

A representation of the gaze regions and the list of hand actions is depicted in Table 5. It took about 7 min for every execution of the gaze protocol, and the length of the resulting recordings is around 3 min.

### 3.3. Multi-Sensor Setup Architecture

The selection of the sensor architecture is one of the main pillars for a successful generation of a CV dataset. These sensors should provide enough data quality to collect the desired information in the form of images. For the case of the DMD, a set of requirements in terms of sensor capabilities were identified that fulfil the overall objectives of creating a dataset for DMS applications. The following requirements were identified:

- **Camera-based sensors:** Data extracted from the sensors primarily relies on a visible feature inside the vehicle and driver.
- **Multi-modal camera sensors:** For a diverse and richer collection of data, the different modalities include RGB images, IR images and depth data.
- **Multi-camera arrange setup:** To capture several characteristics of the driver, multiple views of the inside of the vehicle are required.
- **Synchronized images:** Data from the different cameras and multi-modal streams are timestamped, synchronized and aligned.

Depth sensing has become one additional source of data to improve the accuracy of computer vision tasks. Three depth-sensing modalities are the most relevant: stereo sensors (both active and passive), structured light, and time of flight (ToF). Attending to the defined requirements, we have analysed three main characteristics of sensor performance in order to select the camera used for recording the DMD: range, resolution and reliability.

**Range** is perhaps the most important parameter. In driving monitoring, the cameras shall have a capture range where body parts such as faces, hands and limbs are accurately sensed. Since the person will be inside the car cabin, this limits the range to less than 2 m. Depth sensors have ranges that start at around 0.5–1 m from the sensor and extend to 3–6 m at best. Here, stereo sensors can provide a good trade-off between range and its camera baseline.

Regarding **resolution**, it depends on the range as well as the nature of the optics used by the sensor. Structured light sensors and active stereo sensors’ resolution are limited by the density of the pattern that they project and the resolution of the IR camera. In the DMD, the resolution of the camera sensors is selected to be high enough to capture the smaller parts of the driver’s body (i.e., the eyes and mouth).

In third place, sensors’ **reliability** depend on the inherent limitations of each sensor’s modality. Stereo sensors, for instance, need sufficient ambient light in the visible spectrum to work properly. Conversely, infrared-based sensors such as structured light and ToF suffer when exposed to too much ambient light, making them not appropriate for exterior applications. Active stereo sensors with infrared pattern projectors offer the *best of both worlds*, increasing sensor reliability over a number of environments and lighting conditions.

To build the DMD we chose the Intel^®^ RealSense^™^ as they provide a good trade-off between hardware cost, power consumption and form factor. Models D415 and D435 matched the active stereo sensors requirements for the three modalities (RGB, IR and Depth).

**Figure 4:**
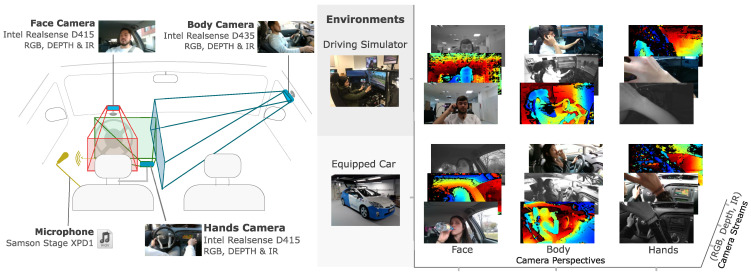
DMD camera setup and recording environments.

We placed three cameras to record the driver’s face, body and hands. The location of the cameras is shown in Figure 4. The selection of the camera model was conducted based on the field of view (FOV) of the available cameras, without compromising the minimum range for depth sensing.

Other in-cabin multi-sensor monitoring architectures published have either focused on single-camera set-ups to monitor the driver or the passengers [56]. While other works have increased the number of cameras for inside sensing to include body sensing cameras [57] or depth information from one view [50], to the best of our knowledge no other works have presented 3 cameras (with 3 modes: RGB, IR and Depth) each of them to monitor different aspects of the driver (body, face and hands).

In Table 6, the most relevant characteristics of cameras D415 and D435 are shown. Model D435 was used to capture the body of the driver due to its larger FOV and D415 was used for face and hands sensing due to its lower price tag.

To extend the completeness of the DMD, a microphone *Samson Stage XPD1* was attached to the driver to record usual sounds during the performed activities. The audio information of the microphone was recorded for future utilization, as a complementary data stream that can help to distinguish actions that may be difficult to label or detect using only image information. For instance, “talking” or “talking to passenger” are actions labelled in the DMD whose start and end time instants could not be easily determined by only looking at the images. For the moment, the audio information is not being labelled because of lack of resources. Extending vision-based datasets with other modalities is beneficial for the multi-modal study of the detection tasks, allowing practitioners from different areas of expertise, besides computer vision, to explore the relationship between visual features and other data modalities.

In addition, two twin recording scenarios were considered: a real car and a driving simulator, as shown in Figure 4. A driver monitoring dataset should be collected in real driving conditions with a real car. However, there are some activities to be recorded which may put at risk the driver’s safety which cannot be performed in a moving car on the roads, such as using a cellphone or driving with a high level of sleepiness. For those cases, the methodology presented in this work defines alternatives that mimic real conditions. Those cases were captured using a stopped car and the driving simulator. The purpose of the driving simulator is to immerse the driver in a close-to-real driving experience. The position of the cameras was placed at the same distance from the driver seat in both the real car and the driving simulator.

### 3.4. Participants Selection

**Figure 5:**
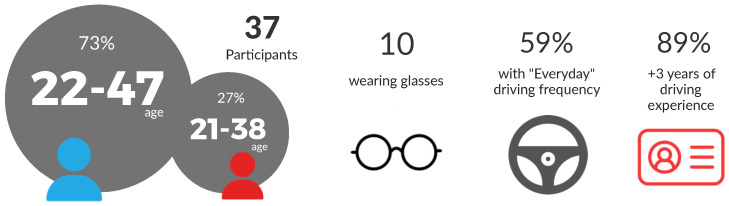
Participants information.

The success of a feature-rich visual dataset highly depends on the variety of characters that appear in the recordings, especially when human participants are the subjects of the analysis. To have a diverse collection of driving monitoring videos, the participants should meet different requirements: diverse ethnics, ages, driving experience and visual characteristics. As shown in Figure 5, for the recording of the first stage of the DMD, 37 driver volunteers participated. This group offers variability in gender: 73% men and 27% women. The age distribution of the participants was kept homogeneous in the range of 21 to 50 years of age. In addition, 10 participants regularly use glasses when driving. These participants were selected to assure novice and expert drivers were included in the recordings. Each participant signed a GDPR informed consent, which allows the dataset to be publicly available for research purposes.

No dataset is perfect (a look at any major dataset may throw non 50–50% balance in many dimensions, e.g., Waymo, Apollo, Mapillary-Vistas, and any of the driver monitoring datasets referenced in Table 1). It is very difficult to reach equilibrium in all aspects, specially when datasets are as complex as the DMD. Context, illumination, variety of actions and subjects may easily scale up the combinations exponentially. This is actually an industrial problem manufacturers and AI practitioners are facing nowadays. We have performed our best considering the limited resources we had to hire additional subjects, and thus had to use volunteers from our institution. However, in our opinion, the balance we have obtained, despite not being perfect, reaches sufficient representativeness to research and build applications with the DMD.

Participants were grouped into 6 groups of 5 individuals and 1 group of 7. Each participant was asked what time of the day they could participate in the recordings: in the morning, in the afternoon, or both. Knowing the availability of the participants, they were organised by groups depending on the recording sessions they could attend. Then, a double participation schedule was assigned to 3 groups of volunteers whose availability was “all day”. All the protocols were recorded twice for these participants. Meaning that the same person did the same activity with morning and afternoon lighting, not on the same day, contributing to variations in clothing. In the DMD, the material of 15 people meets this condition.

Taking into account all these considerations and the estimated times of each protocol, the daily planning of the recordings program was sent to each group with specific dates and times. This is how the participants were organised, making the logistics of the recordings easier in general.

### 3.5. Recording Sessions

Once the preparation stage is completed, the recording sessions can take place. One common problem faced in real cars is the reduced bandwidth support for data transmission due to the limitations in the on-board PC hardware. To overcome this common case in automotive applications, we used a dedicated recording computer capable of supporting intense work and movements in the car. This computer was equipped with 3 dedicated USB 3.0 ports to support the connection interfaces of Intel RealSense cameras. The PC Hardware specifications for the recordings are:

- CPU: Intel i9 7940X series X 4.30 GHz
- RAM: 64 GB
- HD: 2 × SSD 1 TB M2
- GPU: 2 × NVIDIA GeForceTM RTX 2080Ti 11 GB GDDR6 PCIe 3.0

The selected cameras allow the user to configure different parameters to improve the capture quality of the data. For any dataset acquisition task, the dataset designers should pay special attention to the parameters which affect the image quality. When building datasets where multiple cameras are present, different factors affect the performance and the allowed configuration. Intel RealSense cameras can operate in multiple modes for frame resolution and FPS, but these parameters are limited by the bandwidth supported by the connection interface and recording PC. In general terms, the camera’s parameter selection should try to maximize the frame resolution and FPS for all the cameras. Having larger images helps to capture smaller body part regions with more detail, and having larger FPS enables better video quality in fast-moving scenes. For the DMD, more importance was given to the frame resolution, since the driving actions does not imply fast movements (except for blinking).

The frame resolution and FPS were selected attending the recommended allowed data bandwidth of USB 3.0 connections. The USB 3.0 “SuperSpeed” interface of the Intel RealSense cameras supports 5 Gbps. However, considering the encoding overhead, the raw data throughput is 4 Gbps. The USB specification considers it reasonable to achieve 3.2 Gbps. This could be an optimistic upper limit. As the USB protocol can support several devices connected to the same port; another analysis [58] showed that a device that requires over 30% of the bus bandwidth should be considered to be used separately from other devices in a single USB port. So, in general, Intel RealSense manufacturers recommend not to go further than 0.3 × 4 Gbps = 1200 Mbps to ensure robust continuous streaming.

Then, as shown in Table 7 the camera configuration that is closest to this recommended bandwidth and maximizes the frame resolution is for images of 1280 × 720 pixels and 30 FPS in all three modalities. The three selected cameras were plugged into three independent USB controllers to have the maximum bandwidth possible.

Moreover, image adjustments were made to improve the image quality of the recorded images. The selected cameras allow the definition of dedicated regions of interest (ROI) to perform auto exposure adjustment. This step was required to obtain well-lit images of the body of the driver. Then, for each camera, a ROI around the driver’s body parts was configured. The ROI corresponds to the face, body and hands for each camera.

In addition, an important characteristic of multi-modal datasets is the requirement of having synchronized data streams. During the recording sessions, the three-camera streams were recorded using ROS bag format (http://wiki.ros.org/Bags/Format, accessed on 22 March 2022). The advantage of using the rosbag format is the possibility to store the three modalities of the camera in one file, including additional metadata such as camera properties and timestamps. With the timestamps, it is possible to obtain synchronized multi-modal streams. However, rosbag files also add some overhead to the output, which increases the final size of the file and potentially impacts the recording bandwidth. The selected camera stream’s configuration has a bandwidth of 1327 Mbps, which is almost 10 GB per minute of recording per camera. In order to reduce the final size of the rosbag per camera, we applied the lossless compression data algorithm LZ4, which reduces the final size of the videos to approximately 6 GB per minute.

During the recording sessions, after each group session (morning or afternoon) was completed, the recorded files were transferred to centralized network-attached storage (NAS). Although the recording PC was equipped with 2 TB SSD hard drives, the generated data volume of the cameras (ref. Table 7) easily fills the HD. This process was conducted during the break times between recording sessions to avoid processing overhead on the PC.

**Figure 6:**
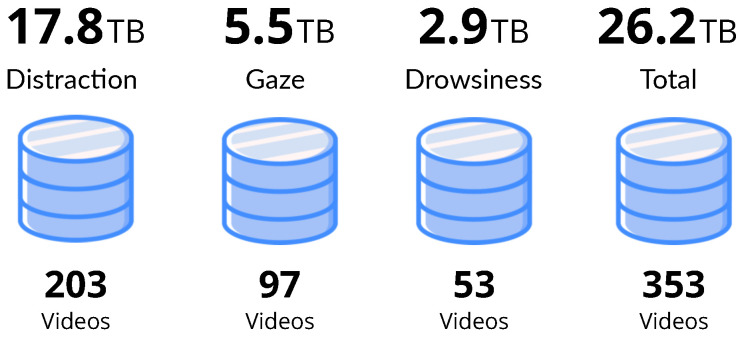
DMD material weight in raw and video count.

The total weight of the material obtained in raw is about 26 TB. The material distribution in relation with how it was captured (the recording scripts presented in Section 3.2) is shown in Figure 6. There are 203 videos that follow the distraction recording protocol presenting drivers performing activities related to distraction, 53 showing signs of drowsiness and 97 videos in which gaze direction and hands position can be analysed.

## 4. Dataset Post-Processing

After the recording session was completed, all the recorded material was located in a centralized NAS. This raw data was kept as backup for future post-processing actions. As the DMD was intended to tackle the lack of data for different driver monitoring tasks, it was crucial to store the material with the highest quality possible since these tasks could require different compression qualities. However, to facilitate the consumption of the visual material by annotation tools and DL training algorithms, a post-processing stage was implemented to export, calibrate and synchronize the multi-camera streams. The images and videos were created using the FFMPEG library (https://www.ffmpeg.org/, accessed on 22 March 2022).

### 4.1. Stream Compression

Raw data is in rosbag format (.bag) to annotate, work with the material and visualize it, it was necessary to extract the data from the rosbag. For this, a Python script with ROS library was developed.

- **RGB and IR:** The images are extracted and saved in PNG format with a size of 1280×720 pixels. After having the total number of images per stream extracted, they are encoded in a video with libx264 encoder and saved in MP4 format with a bit rate of 15,000 kbps and a frame rate of 29.98 FPS for hands and face camera videos and 29.76 FPS for body camera video. These frame rates were the ones stored by the cameras during the recordings.
- **Depth:** Depth data contains information of distance (in millimetres), indicating how far the object is from the camera, or the driver in this case, in every pixel. Therefore, this information can be extracted as an image where each pixel contains a distance value instead of a colour value. This image is in 16UC1 format, which allows each pixel to have a 16-bit value in one channel. This pixel format is equivalent to gray16le.
  To save as an image and preserve this pixel format, depth frames were saved in TIFF format (.tif). After exporting all images, the videos were encoded with FFV1 codec and saved in AVI format. The FFV1 codec is lossless and supports gray16le pixel format (images in 16UC1).
  The resulting video does not have a visualization purpose; the images are not recognizable with conventional video players, they contain distance information. This effort was made to make the distribution task simpler, since the IR and RGB are also distributed in video format.
- **Metadata:** Inside the rosbag, there was also information about the recordings that were extracted and saved in the annotation file. This data is considered in the annotations list as scenario metadata (subject, context, video and camera information), shown in Figure 3.

### 4.2. Multi-Sensor Stream Synchronization

When hardware synchronization is not possible, capturing with multiple cameras produces a small time shift (a few frames) in different streams. In this case, it happened that the 3 cameras operated asynchronously, hence, they did not start recording at the same instant; most of the time, the first camera to switch on and start recording was the face camera, then the body camera and finally the hands camera. Therefore, there is a frameshift between videos in every recording. Manually correcting this shift in every video per recording would require much effort and time. As a solution for this, we created an automatic method to identify those shifts by extracting the “movement” signals of each camera, calculating the absolute difference between consecutive frames from regions of interest of the image. The shifts are given when analysing the correlation of the signals of the corresponding videos.

The intuition behind this is that the movements of the driver have the same pattern independent of the capture perspective.By understanding the normalised absolute difference of the images of two consecutive frames as “movement”, with the analysis of all the frames, we can get signals of “movement” per video. These signals must correlate since they belong to the same moving person.

**Figure 7:**
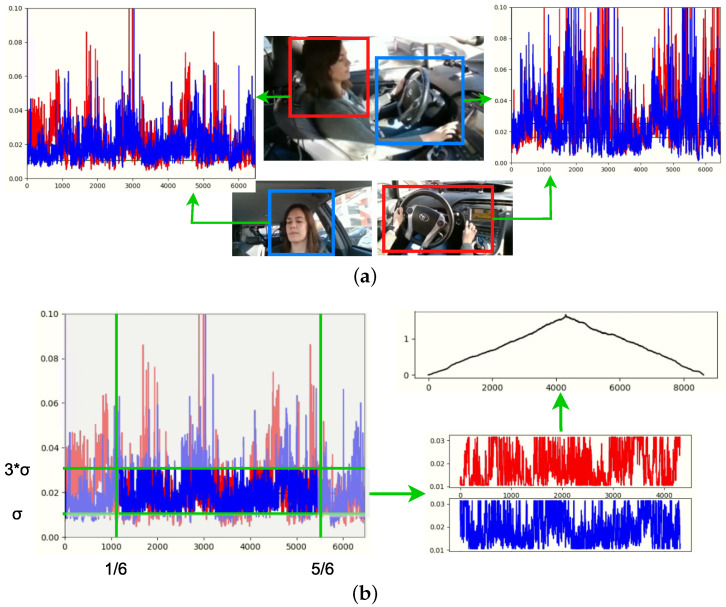
Process for multi-sensor stream synchronization: (**a**) Region of interest and signal extraction, (**b**) Processing of temporal signal and correlation calculation. Blue signal is computed from face and hands ROI in face and body camera, respectively. Red signal is computed from face and hands ROI in body and hands camera, respectively.

To be able to make these comparisons between the 3 cameras (face, body and hands perspectives), some regions of interest (ROI’s) must be established, as shown in Figure 7a. The body camera and face camera are related by the driver’s face, since this is the part of the driver visible from these two perspectives; the hand camera and body camera are related by the driver’s hands. To find the shifts, these ROIs were taken into account to produce signals that could better correlate with each other. There is a signal per ROI and they are correlated depending on their relationship with each other.

It was found that better results were achieved in the correlation result if the signals were passed through a bandpass filter to reduce the signal amplitude. The goal is to get the most overlapping possible between the 2 signals. Some peaks or densities at the bottom of the signal could harm this calculation for the most overlapping rate. To filter the signals, the standard deviation σ was calculated and the band-pass filter was applied from max(x,σ) to min(x,3σ) of the signals’ amplitude. Moreover, the signal was cut 1/6 from the starting and ending sides. This process is shown in Figure 7b.

Then, the frameshift number is defined by the minimum length of the videos minus the point where the highest correlation was reached minus 1. Once the shifts between videos were identified, an additional mosaic video with the 3 streams synchronized was created per recording session. This mosaic video is used in the next phase of the dataset preparation, for semi-automatic label annotation.

### 4.3. Semi-Automatic Labelling

The generation of a visual-based dataset implies careful planning of a concatenation of several steps to finally obtain a collection of visual data and metadata annotations. The recording process, especially when a large amount of data is collected such as in the DMD, has to be performed correctly to make the process of annotating the results easier. That is why we have focused on the definition of the whole framework process of creating a large set of data. In this process, an important part is the generation of metadata annotations. Annotating a large dataset is not an easy task, especially when the projected annotations include multiple dimensions of a computer vision problem, such as in DMS. To annotate the DMD, we applied *semi-automatic labelling* [59] methodology aimed to reduce annotation times. The process consists of the generation of a first set of labels based on weak models. Then, a human-in-the-loop approach is included to provide manual annotations and recursively improve the label’s quality.

For the temporal annotations of the DMD, a tool was developed to make annotation tasks easier and faster to complete (TaTo, Temporal Annotation Tool, version V1 [60]); it is Python-based and allows the visualization of frame-by-frame annotations by colours on a timeline, which makes this process more intuitive. The output metadata was structured using OpenLABEL annotation format [61].

For this work, we extended this tool (TaTo V2) and added some *semi-automatic labelling* principles with new strategies to create some annotations automatically and improve labelling time:

- **Pre-annotations predicted by model:** We only performed manual annotation for the driver actions of a few samples of the DMD with TaTo. These were then prepared to become training material. Then, we trained a model using transfer learning from a Mobilenet [8] originally trained with ImageNet [12]. The predictions were taken as pre-annotations and the annotator’s job was to correct and continue annotating instead of starting from zero.
- **Logical relations among levels of annotations:** There exist some logical relations between levels of annotations that, taken into account, could save time in the labelling process. For example, if the driver is sending a message with their left hand, this should be annotated in the driver_actions level as “texting-left”, but this action also could imply that the person is only using his/her right hand to drive (since he/she is using the left hand to text). Therefore, in the hands_using_wheel level, there should be the “only right” label. Other relations are the talking-related labels as “phonecall-left” in the driver_actions level and “talking” in the talking level. In TaTo, we implemented this function of applying logical annotations; once the annotations from the driver action level were completed, by pressing the “x” key, these logical annotations were propagated to the rest of the levels. This way, the annotator did not have to start annotating from zero.

The TaTo tool, in its second version, offers the advantages of pre-annotations described above. To know the impact of pre-annotations, we measured the time of annotation spent on videos from different recording sessions with both versions of the tool (V1 does not offer pre-annotations). We obtained the times shown in Table 8. Comparing the annotation time per video of each version of the tool, we achieved a decrease in the annotation time with a mean of 66% improvement.

With this, it is shown that semi-automatic annotation strategies can bring advantages over a traditional annotation process. This demonstrates the importance of including semi-automatic strategies during the dataset creation process.

## 5. Discussion

### 5.1. Sample Utilization of the DMD: Action Recognition

Distraction detection is one use case of the DMD. The first approach of distraction detection was made in [62], where the DMD material was annotated with some distraction-related labels that led to the definition of a training dataset called dBehaviourMD. Other iteration in the annotation process resulted in the creation of labels destined for distraction detection that were used in the research of [60]. Recently, thanks to the metadata methodology and sensor configuration defined in this work, we have built a more descriptive and detailed annotation criterion that can support distraction detection through action recognition algorithms. The annotations defined for this specific task are temporal, and the labels, organised by levels, are presented in Table 2.

**Figure 8:**
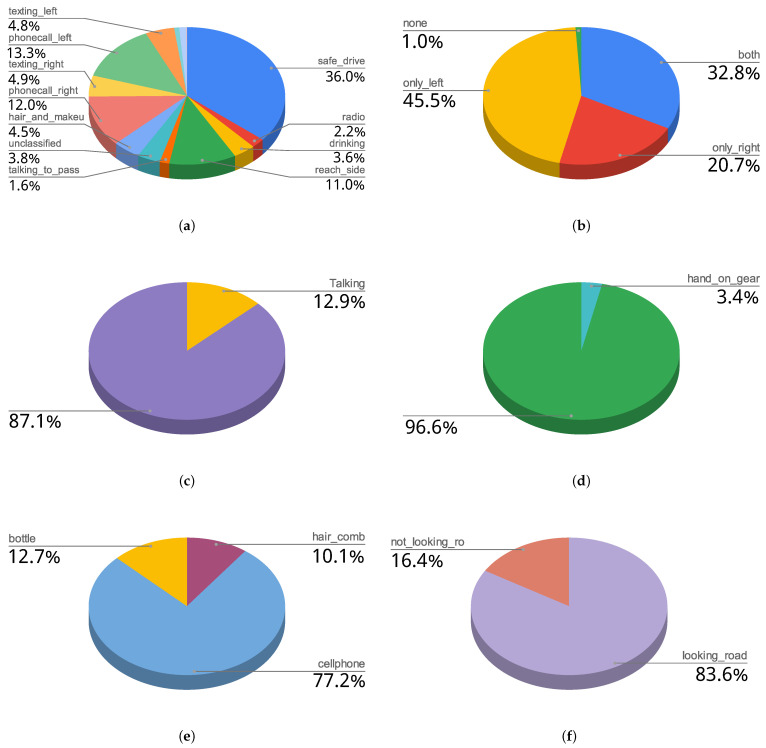
DMD distribution for: (**a**) Driver_actions (**b**) Hands_using_wheel (**c**) Talking (**d**) Hand_on_gear (**e**) Objects_in_scene (**f**) Gaze_on_road.

The label organisation by levels of annotation allows making a multi-label annotation of the video, offering a better description of the scene in one frame. As a result, this temporal distraction-related part of the DMD has the class distribution, per annotation level, presented in Figure 8.

The total frame count of this dataset is 1,837,041 frames, which is about 1021 min of video. The percentages of the labels presented in the table are the percentages of frames annotated with that label within its level of annotation, not with respect to the total amount of frames. For example, for the frames annotated with the “gaze on road” level criteria (frames where the face camera is available), in 84% of them the person is looking at the road and in 16% is not looking at the road.

This part of the DMD is now published on the DMD website (https://dmd.vicomtech.org/, accessed on 22 March 2022) and is free to download under the specified terms of utilization.

### 5.2. Ongoing and Future Work

The construction and preparation of the DMD is part of a live project to build a robust and rich driver monitoring dataset that evolves as the technologies improve [55]. Since there are not many datasets dedicated to driver monitoring, and due to our intention to contribute to the scientific community, the DMD is planned to be published on its website for free to download under non-commercial use agreement. At this point, about 80% of the material related to distractions of the DMD is public, the other 20% will remain unpublished for future benchmarking exercises.

**Figure 9:**
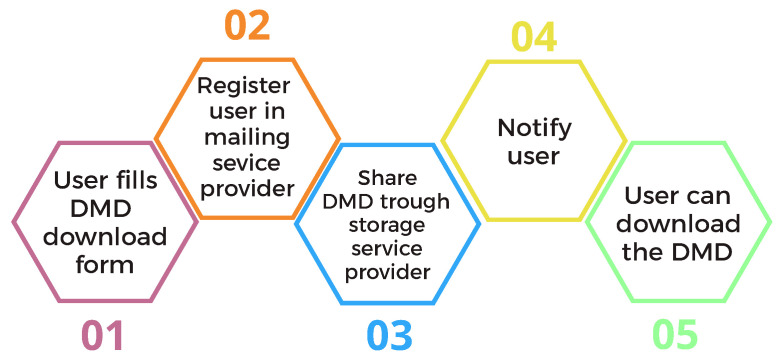
Procedure to distribute the DMD.

A procedure has been developed to allow the user of the DMD to access it easily. The steps for the download process are shown in Figure 9. On the website, there is a download form where the users enter their contact details. This form is sent to a mailing service platform where this information is stored. For each record added, the storage service trigger is activated to give access to the registered email user to the DMD. Once access is given to the storage service, an email is sent to the user notifying them that they can now obtain the dataset. By this time, 418 users have requested the dataset through the website form.

**Figure 10:**
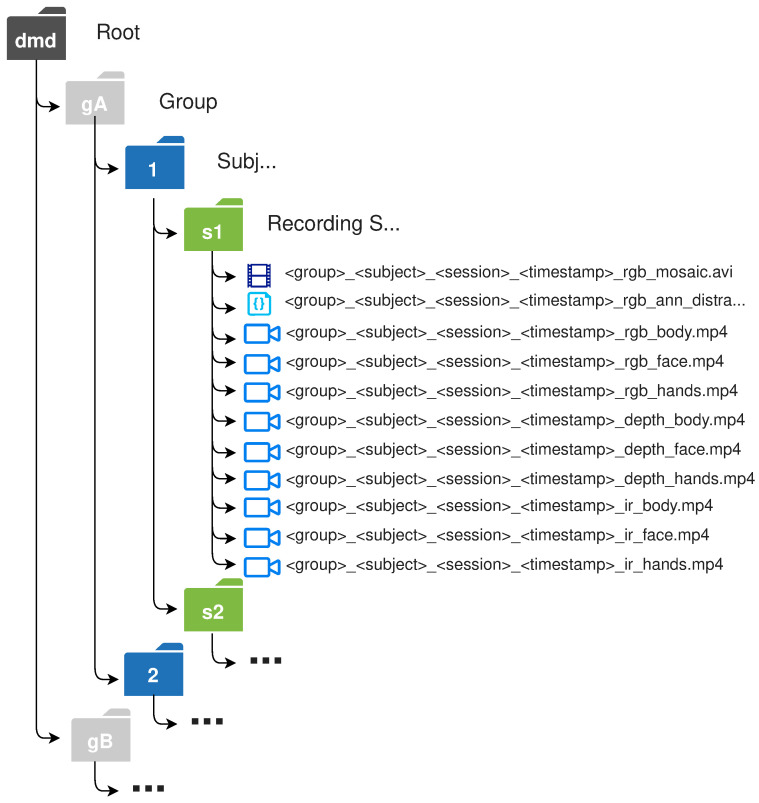
DMD file structure.

An adequate structure to share the dataset was also defined. The material is distributed mainly by groups; the same groups in which the recordings were organised. Then, it is organised in folders by subject to finally group the material by recording sessions. Regarding the nomenclature, each video has in its filename information about the group to which it belongs (A, B, C, etc.), the subject (1, 2, 3 and …), the session (s1, s2, s3 or s4), the timestamp when it was recorded, the information channel (RGB, depth and IR) and the camera (body, face, hands and mosaic) or annotation (ann_distraction). This structure is represented in Figure 10.

As described above, the planned annotations to the DMD is a long and varied list. The objective is to continue progressing in the annotation of the DMD and, in this way, expand the possible uses of the dataset. In turn, the DMD is conceived as a project open to the community so that it can take advantage of it. That is why we continue publishing both material and tools that are developed for the processing and annotation of this dataset.

## 6. Conclusions

Visual data is one of the most important assets to build robust and complete machine vision systems. The process of generating a vision-based dataset is not a trivial and easy task and requires careful planning and execution to avoid redundant work and provide the material that meets the volume, variety and quality requirements of computer vision systems.

In this paper, we presented the full process to build from scratch a multi-modal and multi-sensor computer vision dataset for the application in driving monitoring. We have demonstrated that a previous phase of analysis of the DMS algorithm requirements is needed to define the expected taxonomy of events that are later annotated. In the presented framework, we show it is possible to define several capture protocols that cover the required annotation taxonomy. In addition, based on the recording planning, the selection of the used sensors was specified. For the DMD, the required sensors include several RGB and depth cameras.

Since the DMD was targeted to collect a high amount of driving data, this paper presents the challenges of the post-processing phase, where the raw data was prepared and formatted for consumption by annotation tools and DL training algorithms. To the best of our knowledge, the DMD is the largest and richest public dataset for developing multi-modal video-based driver monitoring systems.

## Figures and Tables

**Table 1:** Comparison of public vision-based driver monitoring datasets.

Dataset	Year	Drivers ^a^	Views ^b^	Size ^c^	GT ^d^	Streams	Scenarios	Usage
CVRR-Hands [48]	2013	8 (1/7)	1	7 k	Hands, Actions	RGB Depth	Car	Normal driving, Distraction
DrivFace [47]	2016	4 (2/2)	1	0.6 k	Face/Head	RGB	Car	Normal driving, Head pose
DROZY [51]	2016	14 (11/3)	1	7 h	Face/Head Physiological	IR	Laboratory	Drowsiness
NTHU-DDD [52]	2017	36 (18/18)	1	210 k	Actions	RGB IR	Simulator	Normal driving, Drowsiness
Pandora [49]	2017	22 (10/12)	1	250 k	Face/Head, Body	RGB Depth	Simulator	Head/Body pose
DriveAHead [53]	2017	20 (4/16)	1	10.5 h	Face/Head, Objects	Depth IR	Car	Normal driving, Head/Body pose
UTA-RLDD [46]	2019	60 (9/51)	1	30 h	Subjective KSS labels	RGB	Laboratory	Drowsiness
DD-Pose [38]	2019	24 (6/21)	2	6 h	Face/Head, Objects	RGB ^e^ Depth ^f^ IR ^f^	Car	Normal driving, Head/Body pose
AUC-DD [54]	2019	44 (15/29)	1	144 k	Actions	RGB	Car	Normal driving, Distraction
Drive&Act [50]	2019	15 (4/11)	6	12 h	Hands/Body, Actions, Objects	RGB ^e^ Depth ^e^ IR	Car	Autonomous driving, Distraction
DMD (ours)	2021	37 (10/27)	3	41 h	Face/Head, Eyes/Gaze, Hands/Body, Actions, Objects	RGB Depth IR	Car, Simulator	Normal driving, Distraction, Drowsiness

^a^ Number of drivers (female/male); ^b^ Simultaneous camera views of the scene; ^c^ h: hours of video, k: image number; ^d^ Ground-truth data; ^e^ only for side view; ^f^ only for face view.

**Table 2:** Subset of Annotation levels for distraction detection in the DMD.

Level	Labels
Camera Occlusion	- Face camera - Body camera - Hands camera
Gaze on Road	- Looking road - Not looking road
Talking	- Talking
Hands Using Wheel	- Both - Only right - Only left - None
Hand on Gear	- Hand on gear
Objects in Scene	- Cellphone - Hair comb - Bottle
Driver Actions	- Safe drive - Texting right - Texting left - Phone call right - Phone call left - Radio - Drinking - Reach side - Hair and Makeup - Talking to passenger - Reach backseat - Change gear - Standstill/Waiting - Unclassified

**Table 3:** Actions performed in the distraction protocol.

Car Stopped	Car Driving	Simulator Driving
Safe driving	Safe driving	Safe driving
Reach object backseat	Operating the radio	Brush the hair
Reach object side	Drinking	Phone call—right hand
Brush the hair	Talk to passenger	Phone call—left hand
Phone call—right hand		Texting—right hand
Phone call—left hand		Texting—left hand
Texting—right hand		Drinking
Texting—left hand		

**Table 4:** Actions performed in the drowsiness protocol.

Car Stopped and Simulator Driving
Drive normally
Sleepy driving
Yawn without hand
Yawn with hand
Micro-sleep

**Table 5:** Actions performed in the gaze–hands protocol.

Car–Simulator–Driving	
Gaze Zones	Hand Actions
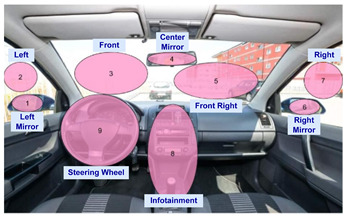	Both hands on (not moving)
	Right hand on (not moving)
	Left hand on (not moving)
	Both hands off (not moving)
	Both hands on (moving)
	Right hand on (moving)
	Left hand on (moving)

**Table 6:** Specifications of Intel^®^ RealSense^™^ cameras used to generate the DMD.

	D415	D435
Use Environment	Indoor/Outdoor	Indoor/Outdoor
Depth FOV (H×V)	$65^{{}^{\circ}}\times 40^{{}^{\circ}}$	$85^{{}^{\circ}}\times 58^{{}^{\circ}}$
Depth Resolution	Up to 1280×720	Up to 1280×720
Depth Frame Rate	Up to 90 FPS	Up to 90 FPS
RGB FOV (H×V)	$69^{{}^{\circ}}\times 42^{{}^{\circ}}$	$69^{{}^{\circ}}\times 42^{{}^{\circ}}$
RGB Resolution	Up to 1920×1080	Up to 1920×1080
RGB Frame Rate	30 FPS	30 FPS
Min. Depth Distance at Max Resolution	∼45 cm	∼28 cm
Ideal Range	0.5 m to 3 m	0.3 m to 3 m

**Table 7:** Camera recording parameters and required bandwidths. The selected specifications for the DMD is marked in bold.

RGB W × H × FPS (24 bits)	Depth W × H × FPS (16 bits)	IR W × H × FPS (8 bits)	Bandwidth 1 Camera (Mbps)	Bandwidth 3 Cameras (Mbps)
1920 × 1080 × 30	1280 × 720 × 30	1280 × 720 × 30	2157	6470
**1280 × 720 × 30**	**1280 × 720 × 30**	**1280 × 720 × 30**	**1327**	**3981**
848 × 480 × 30	848 × 480 × 30	848 × 480 × 30	586	1758
848 × 480 × 60	848 × 480 × 60	848 × 480 × 60	1172	3517
640 × 480 × 30	640 × 480 × 30	640 × 480 × 30	442	1327
640 × 480 × 60	640 × 480 × 60	640 × 480 × 60	885	2654
640 × 360 × 90	640 × 480 × 90	640 × 480 × 90	1161	3484

**Table 8:** Comparison of annotation times using v1 and v2 of TaTo.

Session	TaTo Version	# Videos	Total Time (h:min:s)	Time/Video (h:min:s)	Improvement
s1	V1	4	13:02:00	3:25:00	56.10%
	V2	1	1:30:00	1:30:00	
s2	V1	3	16:10:00	5:36:00	63.69%
	V2	2	4:04:00	2:02:00	
s3	V1	2	2:20:00	1:10:00	81.43%
	V2	3	0:41:00	0:13:00	
s4	V1	2	19:45:00	8:22:00	63.55%
	V2	3	9:10:00	3:03:00	

## Data Availability

The data generated in this study are openly available in https://dmd.vicomtech.org (accessed on 22 March 2022).

## Abbreviations

The following abbreviations are used in this manuscript:

CV	computer vision
DMS	driver monitoring system
DMD	driver monitoring dataset
DL	deep learning
